# Training on Movement Figure-Ground Discrimination Remediates Low-Level Visual Timing Deficits in the Dorsal Stream, Improving High-Level Cognitive Functioning, Including Attention, Reading Fluency, and Working Memory

**DOI:** 10.3389/fnhum.2017.00236

**Published:** 2017-05-15

**Authors:** Teri Lawton, John Shelley-Tremblay

**Affiliations:** ^1^Cognitive Neuroscience Research and Remediation, Perception Dynamics Institute Encinitas, CA, USA; ^2^Department of Psychology, University of South Alabama Mobile, AL, USA

**Keywords:** dyslexia, perceptual learning, plasticity, timing, reading, attention, memory

## Abstract

The purpose of this study was to determine whether neurotraining to discriminate a moving test pattern relative to a stationary background, figure-ground discrimination, improves vision and cognitive functioning in dyslexics, as well as typically-developing normal students. We predict that improving the speed and sensitivity of figure-ground movement discrimination (*PATH to Reading* neurotraining) acts to remediate visual timing deficits in the dorsal stream, thereby improving processing speed, reading fluency, and the executive control functions of attention and working memory in both dyslexic and normal students who had *PATH* neurotraining more than in those students who had no neurotraining. This prediction was evaluated by measuring whether dyslexic and normal students improved on standardized tests of cognitive skills following neurotraining exercises, more than following computer-based guided reading (*Raz-Kids* (*RK*)). The neurotraining used in this study was visually-based training designed to improve magnocellular function at both low and high levels in the dorsal stream: the input to the executive control networks coding working memory and attention. This approach represents a paradigm shift from the phonologically-based treatment for dyslexia, which concentrates on high-level speech and reading areas. This randomized controlled-validation study was conducted by training the entire second and third grade classrooms (42 students) for 30 min twice a week before guided reading. Standardized tests were administered at the beginning and end of 12-weeks of intervention training to evaluate improvements in academic skills. Only movement-discrimination training remediated both low-level visual timing deficits and high-level cognitive functioning, including selective and sustained attention, reading fluency and working memory for both dyslexic and normal students. Remediating visual timing deficits in the dorsal stream revealed the causal role of visual movement discrimination training in improving high-level cognitive functions such as attention, reading acquisition and working memory. This study supports the hypothesis that faulty timing in synchronizing the activity of magnocellular with parvocellular visual pathways in the dorsal stream is a fundamental cause of dyslexia and being at-risk for reading problems in normal students, and argues against the assumption that reading deficiencies in dyslexia are caused by phonological or language deficits, requiring a paradigm shift from phonologically-based treatment of dyslexia to a visually-based treatment. This study shows that visual movement-discrimination can be used not only to diagnose dyslexia early, but also for its successful treatment, so that reading problems do not prevent children from readily learning.

## Introduction

There is no greater educational problem facing our schools than students who have trouble reading. Although estimates vary among researchers, most estimates of the prevalence of reading problems range from 10% (Shaywitz et al., 1998) up to 80% (National Center for Educational Statistics (NCES), 2013), often with diagnoses of dyslexia or reading below proficiency. Students with dyslexia and other reading difficulties, those classified as having a Specific Learning Disorder by the DSM-V (2013), have problems in their ability to read that are disproportionate to achievements in other academic skills. Reading difficulties are prevalent in the United States (U.S.) where 65% of eighth graders and 64% of eighth graders are not proficient in reading (National Center for Educational Statistics (NCES), 2013). Previous studies have shown that reading difficulties in many children can be prevented through early intervention (Schatschneider et al., 2004). Identification of the cognitive skills that predict subsequent reading ability can help identify children at risk for reading problems (Kevan and Pammer, 2009), and following appropriate training may reduce the severity of their symptoms. Since visual motion detection deficits in pre-reading children often predict who will develop reading problems (Boets et al., 2011), it is of considerable interest to investigate the efficacy of a promising, visually-based reading remediation intervention (Lawton, 2000, 2016) to help struggling readers and normal (typically-developing (TD)) students become more effective readers by improving movement discrimination, thereby improving visual timing, attention, reading fluency and memory.

### Visual Timing and Figure-Ground Movement Discrimination Deficits Underlie Dyslexia

Dyslexia, the most common type of reading disorder, is defined as partial alexia in which letters, but not words, may be read, or in which words may not be decoded (word recognition) or encoded (word recall for proper spelling) at normal levels (Hofstetter et al., 2000). Boder (1973) classified dyslexics into: (1) dysphonetics (trouble sounding out words by word attack); (2) dyseidetic (trouble with sight-word recognition and spelling phonetically irregular words such as “laugh” or “should”); and (3) those who are both dysphonetic and dyseidetic. This definition guided the development of the Dyslexia Determination Test (DDT; Griffin et al., 2003) which is used to classify students as normal (TD) or dyslexic, validated (Guerin et al., 1993) using a shorter version of the DDT. Furthermore, students classified as being dyslexic show significant motion discrimination deficits (Eden et al., 1996; Demb et al., 1998; Lawton, 2000, 2007, 2008, 2011, 2015, 2016; Ridder et al., 2001).

The key stimulus attribute needed to detect motion discrimination deficits are direction discrimination thresholds obtained by measuring the contrast sensitivity (CS) for the direction of motion relative to a stationary background (Georgeson and Scott-Samuel, 1999). Only when the direction of motion is discriminated against a stationary background do both dysphonetic and dyseidetic dyslexics, those dyslexic students with pronunciation and/or spelling problems, exhibit a significantly impaired ability to discriminate the direction of motion (Lawton, 2000, 2007, 2008, 2011, 2015, 2016; Ridder et al., 2001). Since direction-discrimination employs inhibitory circuits (Barlow and Levick, 1965), this indicates that dyslexic students may have a developmental deficit in their inhibitory circuits.

### Mechanisms Underlying Impaired Reading Skills

Students who are at-risk for reading failure have deficits that encompass both pronunciation-based and visual processing-based issues (Stein and Walsh, 1997; Stein, 2001). The biological basis of dyslexia (reading difficulties) was for many years assumed to be in the brain regions responsible for the visual perception of text (Hinshelwood, 1917). This theory has largely been replaced since the late 20th century. At present, it is theorized that the core deficit underlying most reading disabilities is an auditory phonological processing deficit (Tallal, 1980; Bradley and Bryant, 1983; Tallal et al., 1993; Stanovich and Siegel, 1994; Torgesen et al., 1994; Snowling, 2001; Shaywitz, 2003; Temple et al., 2003; Dehaene, 2009; Olulade et al., 2013). A careful examination of the neuroimaging studies responsible for this paradigm shift reveals that visual word form areas and other visual processing areas were also implicated in many of these studies. For instance, Shaywitz et al. (1998) states that “Brain activation patterns differed significantly between the groups with dyslexic readers showing relative underactivation in posterior regions (Wernicke’s area, the angular gyrus, and striate cortex) and relative overactivation in an anterior region (inferior frontal gyrus)”. The finding that the striate (visual) processing area is hypoactive in persons with dyslexia is supported in the literature (Livingstone et al., 1991; Eden et al., 1996; Demb et al., 1998; Shaywitz et al., 1998; Temple et al., 2003; Shaywitz and Shaywitz, 2011; Shelley-Tremblay et al., 2011) and reliably co-occurs with abnormal patterns of cortical activity in areas more typically associated with auditory analyses.

The adoption of those in the educational and medical communities of a “brain-basis” for phonological processing deficits as the key abnormal factor in dyslexia is based on a solid foundation of research, but may have had the unintended consequence of placing phonological processing deficits as the *sole* cause of dyslexia, as opposed to a *sufficient* cause. While phonological processing (linguistic strategies) is a reliable and robust predictor of future reading, it cannot fully account for the variance in reading ability and the full range of deficits in struggling readers, instead only predicting approximately 25% of future reading skills (Mann and Liberman, 1984; Wagner et al., 1997). Moreover, improvements in word reading found following auditory interventions to improve phonological processing may degrade over time, 2 years later showing no difference in word reading compared to controls not having the auditory intervention (Wise et al., 2000).

Recent research extends this view to incorporate a visual processing deficit that compromises visual timing, measured using either: (1) rapid automatized naming (Manis et al., 2000; Wolf et al., 2000); or (2) the contrast needed to discriminate the *direction* (Cornelissen et al., 1995; Slaghuis and Ryan, 1999, 2006; Talcott et al., 2000; Hansen et al., 2001; Ridder et al., 2001; Wilmer et al., 2004; Lawton, 2007, 2008, 2011, 2016; Kevan and Pammer, 2008) and *speed* (Eden et al., 1996; Demb et al., 1998; Wilmer et al., 2004) of moving patterns. Slow reading speeds are a hallmark of students with dyslexia and other reading problems that put students at risk for school failure (Lyon et al., 2003; Nicholson and Fawcett, 2007). Children with reading problems are reported to have some combination of spatial (Lovegrove et al., 1980; Cornelissen et al., 1995; Stein and Walsh, 1997; Talcott et al., 2000; Stein, 2001) and/or temporal (Stanley and Hall, 1973; Kimura and Archibald, 1974; Tallal, 1980; Tallal et al., 1993; Temple et al., 2003) sequencing deficits. These spatial and temporal sequencing deficits have been shown to be prevalent in patient reports that words on the page appear distorted, displaced, or crowded together (Atkinson, 1991), often resulting in eyestrain and headaches (Wilkins, 1995). These spatial and temporal sequencing difficulties, found when images are rapidly presented or moving, have been hypothesized to result from neural timing deficits associated with sluggish magnocellular neurons (Livingstone et al., 1991; Stein and Walsh, 1997; Vidyasagar, 1999, 2001, 2012, 2013; Lawton, 2000, 2007, 2011, 2015, 2016; Stein, 2001; Boets et al., 2011), causing deficits in integration of information between magnocellular (“motion”) and parvocellular (“pattern”) neurons in the dorsal stream. A normally functioning magnocellular pathway is sensitive to *low-contrast* achromatic patterns composed of low spatial frequencies (Kaplan and Shapley, 1986; Sclar et al., 1990). All dyslexics exhibit high contrast thresholds for discriminating the direction of moving patterns against stationary background patterns (Lawton, 2000, 2007, 2011, 2016; Ridder et al., 2001), having trouble with figure-ground discrimination, which was found only for dysphonetic dyslexics when movement discrimination was done on no background (Borsting et al., 1996).

It has been proposed that the visual system exploits the dichotomy of a fast magnocellular channel and a slower parvocellular channel for the purpose of selective attention (Vidyasagar, 1999, 2001, 2012, 2013). The human visual system has predominantly two types of retinal neurons that form two different pathways, the parvocellular, or ventral, pathway (for form discrimination), and the magnocellular, or dorsal pathway (for location and motion processing). Receiving predominantly magnocellular input (Livingstone and Hubel, 1988), the dorsal stream, specialized for processing the movement and location of objects in space (Ungerleider and Mishkin, 1982; Livingstone and Hubel, 1988; Felleman and Van Essen, 1991), projects from the primary visual cortex (V1), through visual area medial temporal cortex, and on to the posterior parietal cortex (PPC), a selective spatial attention area (Posner et al., 1984). The PPC provides the input to the dorsal lateral prefrontal cortex (DLPFC), where working memory is encoded, the predominant cortical areas involved in the Executive Control Network (Menon and Uddin, 2010). This is in contrast to the ventral stream which receives both magnocellular and parvocellular inputs as it projects from V1 through area V4 and on to the infero-temporal (IT) cortex, an area specialized in extracting details relating to an object’s shape and color (Ungerleider and Mishkin, 1982; Livingstone and Hubel, 1988; Felleman and Van Essen, 1991). The faster transmission time of the magnocellular neurons projecting predominantly to the dorsal stream is gated via attentional feedback to the striate cortex (Vidyasagar, 1999), which can then be used by parvocellular neurons in the ventral stream as a starting point for deciphering the individual letters (Vidyasagar, 1999, 2001, 2012; Lawton, 2011, 2016). Moreover, feedback in the dorsal stream from MT to V1 improves figure-ground discrimination (Hupé et al., 1998), a task used in reading. Furthermore, feedback from MT has its strongest effects for stimuli of low salience (Hupé et al., 1998), such as the low contrast patterns that maximally activate magnocellular neurons (Kaplan and Shapley, 1986; Sclar et al., 1990). Studies that have questioned the hypothesis that dyslexics have magnocellular deficits (Skottun, 2000; Amitay et al., 2002; Williams et al., 2003) examined a dyslexic’s sensitivity to flicker or high contrast random dot patterns, relative to *no* background pattern, neither of these stimuli being optimal stimuli for activating direction-selective cells (Baker, 1990; De Valois et al., 2000).

Dyslexics have magnocellular responses that were found to be 20–40 ms slower than normal TD observers (Livingstone et al., 1991; Lehmkuhle et al., 1993), being 2–4 times slower than the normal magnocellular lead time of 10 ms (Dreher et al., 1976) that may cause word distortions, described previously (Atkinson, 1991). Parvocellular functioning among dyslexics has been found to be equivalent to that in normal controls, whereas magnocellular function is significantly impaired (Lovegrove et al., 1980; Hansen et al., 2001; Sperling et al., 2006; Kevan and Pammer, 2009; Gori et al., 2014). Some investigators hypothesize that a lack of synchronization in timing between magnocellular and parvocellular activations in dyslexics may prevent effective sequential processing, pattern analysis, and figure-ground discrimination, and hence impede development of efficient reading and attention skills (Stein and Walsh, 1997; Vidyasagar, 1999, 2012, 2013; Lawton, 2000, 2007, 2008, 2015, 2016; Stein, 2001). It is further possible that the dyslexic reader’s deficit in attentional focus (Vidyasagar, 1999, 2012; Solan et al., 2001; Valdois et al., 2004; Facoetti et al., 2006; Lawton, 2016) is another consequence of sluggish magnocellular neurons, preventing the linked parvocellular neurons from isolating and sequentially processing the relevant information needed for reading (Vidyasagar, 1999), and not from the information overload as proposed previously (Stuart et al., 2001).

The degree to which dorsal stream deficits play a *causal* role in reading failure has yet to be established (Boden and Giashi, 2007; Kevan and Pammer, 2008, 2009). Although previous results indicate that there is a relationship between dorsal stream sensitivity and reading skill found in both pre-kindergarten children before reading is learned, and in older children and adults after the emergence of reading, intervention studies targeting dorsal stream function need to be carried out in order to establish a direct causal link from dorsal stream functioning to reading skill (Kevan and Pammer, 2009). In a previous intervention study, Lawton (2011) showed visual movement-discrimination training caused the reading speeds of dyslexic children to increase from 100 to 1000 percent. This magnitude of effect bears careful replication in order to establish that training dorsal stream function may be essential for developing not only reading fluency, but also the attention networks in both dyslexic and normal TD students.

### Timing Intervention Remediates Attention, Reading and Memory

**Figure 1:**
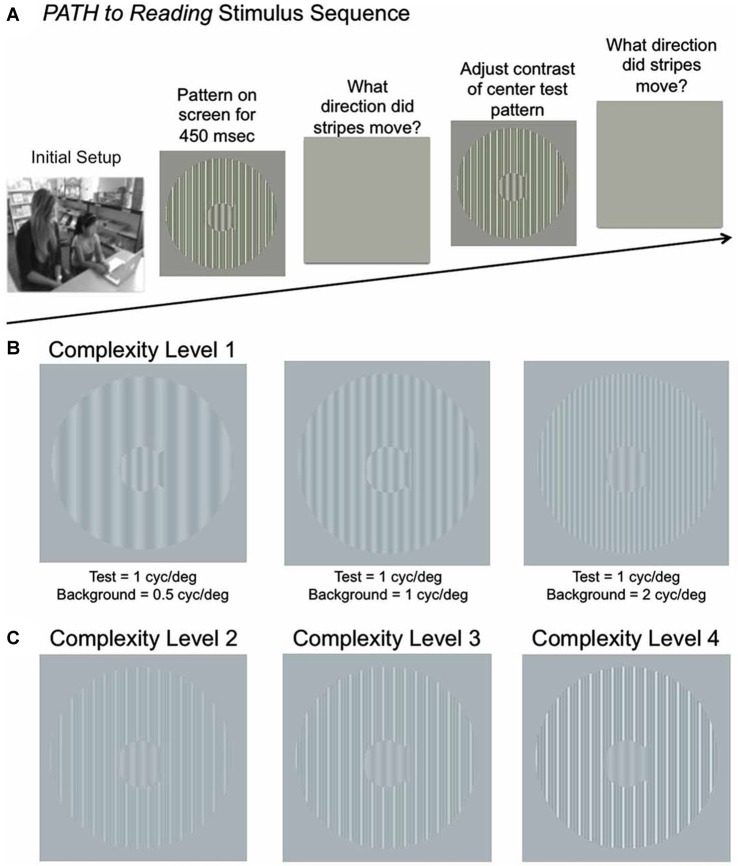
**(A)** Schematic of Stimulus Presentation for *PATH to Reading* intervention. Pattern flashes on screen (shown above) while center stripes move left or right. Screen goes blank, waits for left or right arrow key to be pushed. If incorrect, short tone sounds. Pattern with same or different contrast flashes on screen while center stripes move left or right. Screen goes blank, waits for left or right arrow key to be pushed. This sequence of patterns is presented continuously until the contrast threshold for this pattern is measured. Then the next pattern combination is presented to measure next contrast threshold, until all 20 *PATH to Reading* patterns were presented, and the program says “Thank You” and quits. **(B)** Sample patterns at Complexity Level 1 for a background one octave lower in spatial frequency (0.5 cyc/deg) than the test frequency, equal in spatial frequency to the test frequency (1 cyc/deg), and one octave higher in spatial frequency (2 cyc/deg) than the test frequency for a 1 cyc/deg “fish shaped” test pattern. **(C)** Sample patterns at Complexity Levels 2, 3, and 4 for the center pattern in **(B)**. These patterns have multifrequency background patterns (1 cyc/deg + 2 cyc/deg + 3 cyc/deg) for a 1 cyc/deg test pattern on a 5% (Complexity Level 2), 10% (Complexity Level 3), and 20% (Complexity Level 4) contrast background. These same four complexity levels are repeated at subsequently faster speeds for each set of four complexity levels, increasing from 6.7 Hz (complexity levels 1–4) to 8 Hz (complexity levels 5–8) to10 Hz (complexity levels 9–12) to 13.3 Hz (complexity levels 13–16), as listed in Table 2.

The hypothesis that if sluggish magnocellular neurons contribute to dyslexia, then training to improve the sensitivity and timing of magnocellular processing should improve attention and reading fluency was investigated (Lawton, 2016), using patterns optimal for activating magnocellular relative to parvocellular neurons (Lawton, 2000, 2011). This study investigated whether improving neural timing in the dorsal stream (by improving magnocellular function) improves reading fluency more when training is: (1) in the auditory domain using *FastForWord* (language-based); or (2) in the visual domain using *PATH to Reading (PATH)*, when compared to a traditional reading intervention using linguistic methods that use word building strategies, *Learning Upgrade*, that does not specifically target neural timing. *FastForWord* training lengthens the individual phonemes so that phonological processing improves, the length of the phonemes decreasing as the training progresses. *PATH* training, on the other hand, measures the contrast needed for figure-ground discrimination of sinewave gratings (dim gray stripes) moving left or right relative to a stationary background. *PATH* training employs movement direction-discrimination. These backgrounds increase the task complexity by increasing the number of background spatial frequencies, background contrast, thereby activating more parvocellular neurons, with left-right movement increasing in speed as the training progresses. The patented movement direction-discrimination programs (Lawton, 2000, 2015) were developed based on research into how the brain processes visual information. Visually-based movement discrimination in both normal subjects (Lawton, 1984, 1985) and dyslexics (Lawton, 2000, 2007, 2011, 2016) has demonstrated neuroplasticity in the domain of processing speed using massed practice, i.e., the more students practiced movement discrimination, the more they improved in CS for movement-discrimination, processing speed, and reading fluency. The movement-discrimination training patterns, vertical sinewave gratings (Figure 1), are designed to differentially activate motion-sensitive (magnocellular) neurons in the V1-MT network (Allman et al., 1985; Felleman and Van Essen, 1991; De Valois et al., 2000) relative to pattern-sensitive (parvocellular) neurons, thereby being an effective training stimulus to improve magno-parvo integration deficits at both early and higher levels of motion processing. Unlike movement-discrimination training using vertical sinewave gratings, direction-discrimination using motion coherence which differentially activates motion-sensitive neurons only in MT and at higher processing levels (Zohary et al., 1994; Braddick et al., 2001) has not been shown to be an effective training paradigm (Solan et al., 2004).

Significant improvements in student’s attention, reading fluency, and working memory skills were *only* found following movement direction-discrimination training (*PATH* neurotraining), compared to *Learning Upgrade* (Lawton, 2016), the control intervention. If phonologically-based timing deficits underlie dyslexia, then *FastForWord* should also have significantly improved these academic skills, since training was completed for 50 h (30 min five times/week for 20 weeks) using clever, engaging auditory exercises for twice as long, compared to movement-discrimination training for 20 h (20 min three times/week for 20 weeks). Students trained on movement-discrimination improved significantly more than controls (Lawton, 2016) in attention, reading fluency, phonological processing and both visual and auditory working memory. In contrast, training auditory phonological processing did not improve reading or attention significantly more than found for age-matched controls who were trained on *Learning Upgrade*, suggesting dyslexia is not caused by phonological deficits.

Moreover, all dyslexic students in this study (Lawton, 2016) had abnormal visual motion processing, as found in previous studies (Lawton, 2000, 2007, 2011, 2015). Movement-discrimination CS improved significantly only for those students who were trained on the movement direction-discrimination intervention, improving in sensitivity 3-fold after movement-discrimination training. Not only was CS for movement-discrimination increased significantly following motion training, but also the time to discriminate the direction of motion was reduced significantly for students who were trained on movement-discrimination. That is, the mean time to complete movement-discrimination training decreased as complexity level increased. These results show that as the amount of training increased, both: (1) the sensitivity to discriminate movement-discrimination increased; and (2) the time required to complete movement-discrimination training decreased. The previous study (Lawton, 2016) suggests that visual timing deficits underlie dyslexia. This study intends to extend these results to normal, TD students who also may have visual timing deficits.

### Rationale

The movement direction-discrimination intervention (*PATH* neurotraining) is believed to change the timing of neural responses to be faster (Lawton, 2000, 2011, 2015, 2016) via intensive training of the dorsal stream, improving magnocellular relative to parvocellular activity, thereby improving inhibitory circuits, based on the data on neural plasticity. This theory is based on the idea that the synchronous firing of neurons controls communication in the brain between different areas (Buzsáki, 2006). If the neurons in one area are “sluggish” with respect to the neurons in another area, then they will be unable to synchronize, processing speed will be slowed down and communication, and hence learning, will be compromised. By extensive training on movement direction-discrimination, but not on guided reading, we hypothesize that we are improving the attention and reading networks by increasing the synchronous firing of neurons, thereby increasing the timing and activity of inhibitory circuits.

In this study, two interventions are compared, one targeting the temporal dynamics (timing) of the visual pathways, visual movement direction-discrimination using patterns optimized for low- and high-level dorsal stream processing, with a second reading intervention (control group) using a computer-based guided reading program, targeting high-level cognitive functioning to evaluate whether computer-based neurotraining (*PATH to Reading*), for 20 min three times/week for 12 weeks, improves executive function, attention, working memory, and speed of reading in both dyslexics and normal students, more than computer-based guided reading (*Raz-Kids* (*RK*)) for 30 min three times/week for 12 weeks.

For all children in grades 2–3, both dyslexic and normal (TD) students, this study compares the effectiveness of the movement-discrimination intervention, one that was shown to produce significantly larger improvements in the dyslexic’s attention, reading, and working memory in a previous efficacy/replication study (Lawton, 2016), to *RK*, a computer-based guided reading intervention, typically used in San Diego Unified School District (Business-As-Usual) to remediate reading difficulties. By examining both dyslexic and normal students, this study aims to improve cognitive functioning in a wider range of students than in the previous study (Lawton, 2016). By using *RK*, a computer-based reading intervention, to compare with a visual timing intervention, this controlled for the Hawthorne effect, requiring equal attention for each task. Since *RK* required the student to not only read the words in the stories, but also answer questions about the story to progress to the next chapter, *RK* required more attention from the student than did the movement-discrimination training, and was directly related to improving reading fluency, so it was chosen for the comparison reading intervention. As we have shown above (Lawton, 2000, 2011, 2015, 2016), there is strong theoretical and empirical support for the movement direction-discrimination intervention improving cognitive functioning in both dyslexics and normal observers. Having the entire class participate is required to conduct this study during the school day before guided reading. Moreover, it enables studying normal students, as shown in previous studies (Lawton, 2000, 2007, 2008, 2011) improve in reading speed after training on movement-discrimination twice a week for 12 weeks. We have argued that movement-discrimination neurotraining is not only very effective in improving reading fluency and attention, but it is also a very rapid intervention in terms of the investment of time, making it a practical approach for use in the public schools. The relatively large gains found in earlier studies are promising, and justify investigating movement-discrimination training in this study when compared to a computer-based guided reading task (*RK*) for both dyslexic and normal students. We predict that not only dyslexic but also normal (TD) students who are trained on movement-discrimination will improve in reading fluency, phonological processing, attention, and both visual and auditory working memory significantly more than normal or dyslexic students trained on computer-based guided reading.

## Materials and Methods

All methods were carried out in accordance with the University of California at San Diego (UCSD) guidelines and regulations for scientific studies on human subjects, approved previously by the UCSD IRB (Lawton, 2016). Since these methods were part of the curriculum for every student in the second and third grade classroom, and only short 5–10 min standardized tests of cognitive abilities were administered before and after the 12 weeks of intervention training, this study was exempt from requiring informed consents. Since all children in the classroom participated, this study was part of the curriculum, not requiring informed consents under Exclusion 2. All procedures were conducted in accordance with the tenets of the Declaration of Helsinki.

### Participants

The entire second and third grade at *Innovations Academy*, a public charter school in San Diego, participated in this study. A total of 42 students in grades 2 and 3 (see Table 1), at *Innovations Academy* completed this study, 21 were trained on movement-discrimination (12 dyslexic, 9 normal or TD) and 21 on *RK* (10 dyslexic, 11 TD). Only seven students in these classrooms were excluded, not being included in the numbers of students shown in Table 1, since they were absent from school for too many days during the intervention training; one moved away, three children in grade 2 and three children in grade 3 missed more than 3 weeks of school during the 12 weeks of this study (over 25% of the training), preventing them from completing the intervention training. Whether a student was TD (normal or above normal) or dyslexic was determined by the DDT classification. *Innovations Academy* has 20% special needs students, compared to San Diego Unified School District, having only 5%–7% special needs students.

**Table 1:** **Number and type of participants in study**.

Student type	Dyslexic		Normal	
Intervention type	*PATH to Reading*	*Raz-Kids*	*PATH to Reading*	*Raz-Kids*
Grade 2	3 girls, 3 boys	3 girls, 2 boys	3 girls, 2 boys	4 girls, 2 boys
Grade 3	3 girls, 3 boys	3 girls, 2 boys	2 girls, 2 boys	4 girls, 1 boy
Total number	12 students	10 students	9 students	11 students

This study compared movement-discrimination training to training in a contrast condition, *RK*, a computer-based guided reading program (Business-As-Usual), all students doing a reading intervention to control for the Hawthorne effect. Therefore, no students were put into a group that did no intervention. For 12 weeks, half the second and third grade classes were trained on movement-discrimination and half were trained on *RK* for a total of 30 min twice a week. To prevent intervention crossover, different computer labs were used for training each intervention, except for 3 weeks at the end of the study when the computer labs were being used for school-wide testing, then the training was completed in the multipurpose room. The intervention training was the first activity in the school day, being done right before guided reading in the classroom. Intervention training before guided reading provided each child ample opportunity to practice reading immediately after the reading intervention, as the last efficacy study (Lawton, 2016) found was essential for a high fidelity implementation.

Before and following the 12 weeks of training, all standardized tests were administered using paper test sheets and materials to all students in the study to measure improvements in cognitive and reading skills. Staff administering the standardized tests to students were blind to the type of intervention training that was practiced, since there were so many students in the study. Moreover, staff were not able to discern whether the student improved on cognitive skills, since the initial scores were not available to our staff when doing the final testing, thereby not being able to influence the results. The DDT (Griffin et al., 2003) was used to create two balanced samples stratified in reading ability, and then students were randomly assigned to one of the two reading interventions. Other standardized tests of reading fluency included the *Gray Oral Reading Test* (GORT-5) to measure reading comprehension, a computer-based reading speed test, *Comprehensive Test of Phonological Processing* (CTOPP) Blending Words subtest, *Cognitive Assessment Systems* Stroop and Number Detection Attention tests, *Test of Information Processing Skills* (TIPS) to measure auditory and visual working memory, and the first session of movement-discrimination training to measure visual timing deficits before and after intervention training. The first author and her staff of six UCSD undergraduate Research Assistants (RA) were in charge of supervising the movement-discrimination training. At the end of the study when all data had been collected, the lead RA entered the scores from the standardized test sheets into Excel spreadsheets, where the intervention type was encoded as a number, thereby not having the group type influence the data entry. Subjects were assigned as numbers and not names to prevent any influence over the data collection or analysis. The classroom teacher was in charge of supervising the *RK* training, but the RAs helped supervise both types of training when needed. Since all children in the classroom participated, this study was part of the curriculum, not requiring informed consents under Exclusion 2. The statistical analyses were completed by the second author, so that the first author had no influence over the results obtained.

### Interventions

#### Visual Timing Training Task: (Left-Right Movement Discrimination)

Students in the movement-discrimination training intervention group were instructed by watching a 4-min QuickTime movie (can be viewed by watching student *Motion* movie at www.pathtoreading.com/demo.htm) augmented by verbal instructions from the RA when needed. The student sits in front of a computer monitor with a display similar to the ones in Figure 1, showing low contrast gray stripes. Movement-discrimination training uses displays comprising a stationary, central, fish-shaped window consisting of dim gray stripes moving left or right very briefly (450 ms) that are surrounded by stationary, vertically oriented bars. Test gratings moved 1/4 cycle on each frame, which is the optimal amount of movement for direction-discrimination (Lawton, 1984). The student reported which way the stripes in the fish-shaped window moved by pushing the left or right arrow key. A brief tone was presented after incorrect responses. The program adaptively changed the contrast of the display (making the difference, the contrast, between the white and dark bars bigger or smaller) in order to keep the student at about 79% correct (contrast threshold) using a staircase procedure (Wetherill and Levitt, 1965)—so the student was always doing well, but was always challenged to improve. This staircase procedure provides the most sensitive, repeatable measurements of CS (Higgins et al., 1984). A full training cycle of the movement-discrimination task (two sessions) required 20 threshold determinations (i.e., one for each of the four test spatial frequencies, 0.25 cyc/deg, 0.5 cyc/deg, 1 cyc/deg, and 2 cyc/deg, paired with each of the five background spatial frequencies, being equal to the test frequency or ±1 or 2 octaves from the test frequency), taking between 15–25 min.

There were also levels of difficulty introduced by making the background pattern more similar to that in the fish (test frequency = background frequency), or increasing the background contrast, so that the background recruits more pattern cells, enabling cortical ventral and dorsal streams to be synchronized by working together, and by slowly increasing the speed of movement. Movement direction-discrimination training (*PATH to Reading*) has 16 levels of complexity (see Table 2), for both one and two directions of movement, these two tasks being done sequentially. Movement-discrimination training has many motivational strategies that are implemented to teach the student to learn the task quickly. The student was awarded a score after each contrast threshold was determined, the lower the contrast needed to discriminate movement, the higher is the student’s score. The student earns a star for each complexity level completed. The student catches a fish in the fishnet for every pattern where the motion is discriminated correctly at low contrasts, i.e., those below 1% contrast, accumulating up to 10 fish for the 10 patterns in each session. This is a new motivational strategy to improve the student’s ability to see motion at low contrasts, one that was not used previously. New Telly-Award winning training videos were created to teach this new motivational technique aimed at getting fish in the fishnet.

**Table 2:** **Stimulus characteristics at each complexity level**.

Complexity level	Pattern speed	Background frequencies	Background contrast
1	6.7 Hz	Single frequency	5%
2	6.7 Hz	Multifrequency	5%
3	6.7 Hz	Multifrequency	10%
4	6.7 Hz	Multifrequency	20%
5	8 Hz	Single frequency	5%
6	8 Hz	Multifrequency	5%
7	8 Hz	Multifrequency	10%
8	8 Hz	Multifrequency	20%
9	10 Hz	Single Frequency	5%
10	10 Hz	Multifrequency	5%
11	10 Hz	Multifrequency	10%
12	10 Hz	Multifrequency	20%
13	13.3 Hz	Single Frequency	5%
14	13.3 Hz	Multifrequency	5%
15	13.3 Hz	Multifrequency	10%
16	13.3 Hz	Multifrequency	20%

The stimuli used for training on left-right movement-discrimination were previously found to be optimal for discriminating the direction of movement at low contrasts (Lawton, 1984, 1985, 1989). In addition to the simple backgrounds shown in Figure 1 (complexity level 1), more complex backgrounds were used in combinations that have been found to facilitate movement-discrimination in normal, TD observers (Lawton, 1985, 1989, 2011, 2015). For multifrequency backgrounds, the first background frequency equaled the spatial frequency of the single frequency background, having two additional background frequencies with a difference frequency equal to the test frequency, as shown to be optimal for movement direction-discrimination previously (Lawton, 1985, 1989, 2011, 2015). The complexity level increased when the mean contrast threshold for the 2 cyc/deg test frequency was 1.0% contrast or lower, or after three replications at the same complexity level. Increasing the complexity level increased the: (1) number of sinewave components in the background from one to three; (2) background contrast from 5% to 10% to 20%; and (3) pattern’s speed of movement after every four complexity levels, increasing from 6.7 Hz up to 13.3 Hz, as shown in Table 2. The background contrast was increased to 20% contrast to provide a background that increased parvocellular activity, since magnocellular neurons saturate at 10% contrast (Kaplan and Shapley, 1986). The 20% contrast background required students to analyze information from magnocellular activity relative to increased parvocellular activity, making the task more challenging. The order of presentation for each complexity level was chosen to gradually increase the difficulty of the task (Lawton, 2011). Therefore, as the level of complexity increased, the contrast threshold was expected to be higher. Once all 16 complexity levels of the *Motion* program were completed, the student progressed onto the next program, the *MotionMemory* program. Instead of discriminating the direction one pattern moved by pushing the left or right arrow key as in the *Motion* program, *MotionMemory* required signaling the direction that two separate patterns moved, one after the other, using the four arrow keys to signal which one of four movement directions (right-right (up-arrow), right-left (right arrow), left-right (left arrow), or left-left (down arrow)) was seen. If the second program was too difficult for the student to learn, i.e., not learned after 1 week, then the student was retrained on the *Motion* program, beginning at complexity level 10, so a 10 Hz speed of motion was used for the initial training, as done in the previous efficacy study (Lawton, 2016). The same 16 levels of complexity used in the Motion program, were also used in the *MotionMemory* program, increasing the level of complexity only when the current level was learned or three replications were completed. Each training cycle of movement direction-discrimination took about 15–30 min, and consisted of 20 contrast thresholds. Each threshold required 20–40 trials to complete. A score was given to make the intervention therapy more game-like. The lower the contrast threshold, the higher was the score, and if below 1% contrast earned a fish in the fishnet. Movement-discrimination was trained for either one training cycle or 30 min (pushing “Q” to quit in the middle of a session, where movement-discrimination training resumes the next time) two times a week for 12 weeks. Even though students doing direction discrimination training were given 30 min to complete this training, they only did one training cycle which usually only took 10–20 min.

##### Fidelity of implementation

All contrast threshold data with date and time stamps was stored in individual and summary files, and collected automatically by the computer. Therefore, there was no means for tampering with the data collection. Data in summary files showed the RAs each student’s contrast thresholds, enabling manual overrides of complexity level (e.g., only one pattern prevented the complexity level from increasing automatically). Having the student accrue fish, one for each pattern, showed whether the student was doing the task correctly. Having the student earn a star for each level of complexity on their star sheets let them follow their training progress. The test site, *Innovations Academy*, was monitored by Dr. Lawton on a daily basis to ensure that RAs were supervising learning-impaired students having difficulty doing movement-discrimination correctly, i.e., not getting fish in the net. If low contrast thresholds were not being measured, then RAs who were trained in developing new strategies helped students learn the task more easily. This extra supervision was only needed until the student learned how to do the direction-discrimination task, only being needed in the first few weeks for all but one student. The staff’s main task was to make sure students did not distract their neighbors and focused on the task at hand for both groups.

#### Computer-Based Guided Reading (*Raz-Kids*) Intervention

*RK* was trained for 30 min two times each week for 12 weeks. In *RK*, the student chose, from over 400 e-books, a book to read on the computer at their reading grade level. The computer kept track of where they were in regards to both reading the book while the student viewed the words in the text being highlighted, either visually or auditorily, at a speed the student chose. After each chapter, the student took a comprehension test provided by *RK*, earning stars for each correct answer. The student could not progress to the next chapter unless they passed the comprehension test for the chapter they just finished reading.

##### Fidelity of implementation

All the data from *RK* was recorded automatically by the computer and uploaded to the computer’s database, so that student performance could be tracked by the classroom teacher. A wide variety of reports were available to assist in the analysis of learning profiles of individual students. UCSD RAs and the classroom teacher ensured that *RK* was being administered correctly, ensuring students were connected to the internet, focused on reading their chosen book, and passing comprehension tests at the end of each chapter.

### Measures Used Before and After Intervention

The following standardized tests of academic achievement in reading, attention and memory skills were administered to every student by trained UCSD RAs, at the beginning and end of 12 weeks of intervention training to provide a standardized measure of the improvements in high- level cognitive functions after intervention training. These tests were chosen because they are the “Gold Standard” tests for fast and accurate measurements of fluency-based reading, attention, and memory (both visual and auditory) skills, each test measuring a different cognitive skill.

The standardized tests, all available by typing in their name on the internet, were:

1. *Dyslexia Determination Test (DDT)*. The DDT (Griffin et al., 2003) is the only diagnostic test available that provides a measure of the type and severity of dyslexia; depending on how many words are written correctly, either phonologically (dysphonetic) or spelled correctly (dyseidetic) at each grade level, the child is classified as markedly below normal (1), moderately below normal (2), mildly below normal (3), borderline (4), normal (5), or above normal (6) within 10 min. The DDT was used to determine whether a student was classified as normal (5–6) or dyslexic (1–4).
2. *Gray Oral Reading Test (GORT-5)* to measure Oral Reading Rate and Reading Comprehension, reliability coefficients exceed 0.90;
3. The *Comprehensive Test of Phonological Processing (CTOPP)*: Blending Words subtest where the child combines two or more sounds, provided by a CD, into one word to measure phonological processing, takes about 5 min; reliability coefficients exceed 0.80;
4. *Cognitive Assessment Systems test of Expressive Attention* (*Stroop: color-word interference*) and *Number Detection*, matching particular numbers having a certain font, to obtain a standardized attention score in 10 min; reliability coefficients are 0.92;
5. *Test of Information Processing Skills (TIPS)*—in both visual and auditory modalities to measure sequential and nonsequential short-term and working memory, takes about 10 min; reliability coefficients are 0.89 except for delayed recall which is 0.91;
6. A *Computer-Based Reading Speed* assessment, described previously (Lawton, 2000, 2007, 2011) where six words of white text (0.5 cm wide by 0.5–0.75 cm high) *sans-serif* letters on a black background were presented from subsequent portions of an interesting story at increasing speeds. Just six words were displayed at a time so that: (1) there was no crowding from adjacent words above or below the line being read; and (2) at least two saccades were required to read each line of text. Students were instructed to do the *ReadingRate* task by watching a 4-min QuickTime movie (the movie can be viewed at www.pathtoreading.com/demo.htm). Text at their reading grade level was used to measure reading speeds. Reading speed, measured in words per minute using a double staircase procedure, was not limited by the child’s rate of speaking, as is the case for the GORT above. In addition, words per minute (words/min) is a much higher resolution scale, than the 1–5 scale used to score reading rates on the GORT. Usually, two reading rate thresholds were measured.
7. Contrast Sensitivity for left-right movement discrimination (*Timing Diagnosis* program). Students were instructed to do this task by watching a 4-min QuickTime movie (the movie can be viewed by watching the “student Motion” movie at www.pathtoreading.com/demo.htm).

All of these cognitive assessments, which were age-appropriate, took about 1.5 h to complete. Measuring significant improvements in these measures at the end of intervention training was used to determine whether this training was effective in improving different cognitive skills.

### Statistical Analyses

Change in test performance for the primary and secondary outcome measures (attention, reading speed and comprehension, visual and auditory working memory, phonological processing and movement discrimination) were analyzed using ANOVAs comparing standardized test percentiles, controlling for age, before and after the reading interventions for the two reading interventions, computed for dyslexic and normal (TD) students. Data was pooled across the second and third grade classrooms to provide approximately 10 students in each group, which was sufficient for obtaining statistical significance. The mixed factors ANOVAs were performed with the between subjects factors of Training Group (*PATH* vs. *RK*), and Reading Level (Normal vs. Dyslexic) and the within subjects factor of Training (Pre vs. Post). One analysis was performed for each standardized test described above to compare improvements for *RK* compared to improvements for *PATH* to determine whether movement-discrimination training improved the primary and secondary outcome measures more than found for *RK* training. There were an approximately equal number of boys and girls in each group in each classroom, as shown in Table 1. The ethnicity was also equally distributed among groups. A MANOVA was computed at the beginning of the study to ensure the groups were matched samples.

A two-factor ANOVA without replication was used to compare mean CS for test frequencies 0.25, 0.5, 1.0 and 2.0 cyc/deg at each of the 16 complexity levels within each treatment group, dyslexic or normal (TD), doing movement-discrimination training. A two-factor ANOVA, complexity levels × student type (dyslexic or normal), was used to compare the mean duration needed to complete five contrast threshold measurements for the most sensitive test pattern, i.e., 1 cyc/deg, for dyslexic and normal students at each complexity level. All analyses were performed using ANOVAs and MANOVAs in SPSS 23.0 (IBM).

## Results

This study examined 22 children in second grade, 8.1 ± 0.2 years of age, and 20 children in third grade, 9.0 ± 0.2 years of age, both grades having 55% girls and 45% boys (see Table 1), half the students being dyslexic (22) and half being normal (20). Even though participants were randomly assigned from the ordered list of DDT scores (using numbers from 1 to 6, that go from markedly below normal to above normal, adding together spelling and pronunciation scores) into two groups (movement direction-discrimination or guided reading), the groups were balanced on the variables of age and baseline reading rate, phonological processing, attention and working memory, as shown in Table 4A, and verified using a MANOVA for independent samples to test for differences between the initial standardized test scores (percentiles) for the movement-discrimination group compared to the guided reading group. The multivariate, and also each univariate test from the MANOVA indicated that on all key variables, the two groups were not significantly different (*p* > 0.05). Moreover, there were no significant differences between Dyslexic and Normal readers on any standardized measure at the beginning of this study, except for Reading Rate, *F*_(1,40)_ = 5.624, *p* = 0.023, as determined using a MANOVA with a between subjects factor of group (Dyslexic or Normal) on these indicators. The ethnic distribution for students was 76% Caucasian, 19% Hispanic and 5% Asian.

**Table 3:** **Significance of improvements in contrast sensitivity for each test frequency for dyslexic and normal students**.

Student type	Test frequency	Mean increase per complexity level	*F*_(15)_ value	Significance level *(p)*
Normal	0.25 cyc/deg	15% ± 6%	3.79	0.0001
	0.5 cyc/deg	14% ± 3.9%	4.81	<0.0001
	1 cyc/deg	18% ± 5.7%	3.28	0.0005
	2 cyc/deg	7% ± 3.6%	2.35	0.0099
Dyslexic	0.25 cyc/deg	6% ± 2.3%	2.76	0.0015
	0.5 cyc/deg	11% ± 3.5%	2.79	0.0014
	1 cyc/deg	28% ± 10.5%	2.06	0.019
	2 cyc/deg	19% ± 9.6%	2.53	0.004

**Table 4A:** **Mean ± SEM values for standardized measures in normal students**.

Skill	Training	Measure	Mean pre	Mean post	Diff	Sig
Motion VPL	*Raz-Kids*	VPL	2.6 ± 0.4	3.1 ± 0.5	0.5	ns
	*PATH*		1.4 ± 0.2	5.1 ± 0.3	3.7	***
Reading rate	*Raz-Kids*	Words/Minute	219.4 ± 28.7	223.8 ± 39.7	4.4	ns
	*PATH*		195.2 ± 18.8	303.4 ± 46.1	108.2	***
Comprehension	*Raz-Kids*	GORT %	51.2 ± 9.3	61.1 ± 5.5	9.9	ns
	*PATH*		40.8 ± 9.2	77.8 ± 5.5	37.0	***
Phonological	*Raz-Kids*	CTOPP %	53.3 ± 6.1	60.6 ± 4.4	7.3	ns
	*PATH*		52.1 ± 6.6	71.6 ± 3.8	19.5	**
Attention	*Raz-Kids*	CAS %	33.1 ± 5.1	52.1 ± 7.1	19.0	***
	*PATH*		22.9 ± 6.9	45.4 ± 8.2	22.5	***
Working memory	*Raz-Kids*	TIPS Visual %	61.6 ± 9.9	59.4 ± 10.2	−2.2	ns
		TIPS Auditory %	52.3 ± 9.2	61.6 ± 7.0	9.3	ns
	*PATH*	TIPS Visual %	68.3 ± 6.6	91.2 ± 3.6	22.9	***
		TIPS Auditory %	52.8 ± 11.3	78.6 ± 8.4	25.8	***

### Improvements in Motion Figure-Ground Discrimination

**Figure 2:**
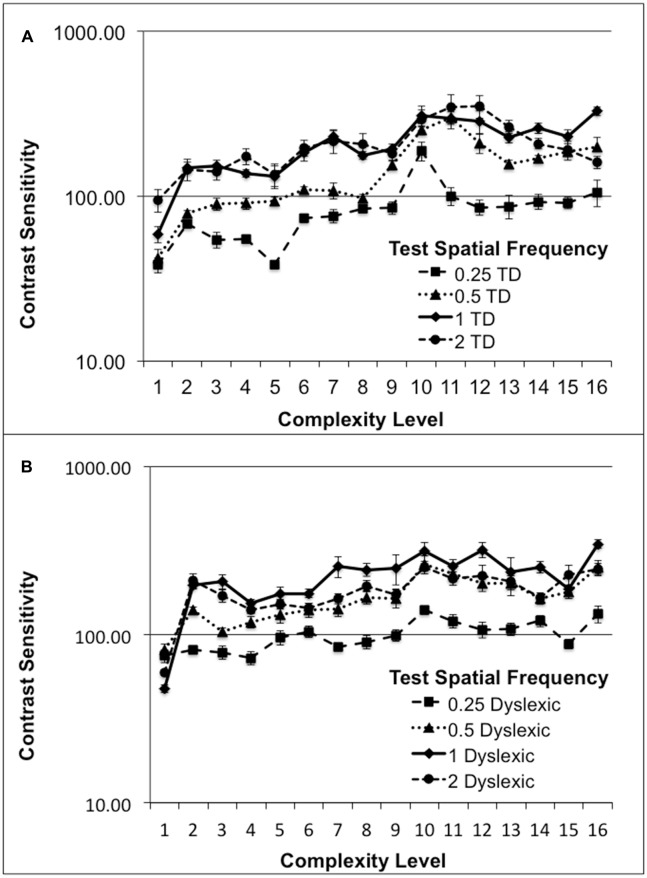
**Mean contrast sensitivity (CS) functions for five normal (typically-developing (TD)), (A)**, and seven Dyslexic, **(B)**, students at each complexity level.

Although, normal students improved more than dyslexic students in their CS for figure-ground motion discrimination, this difference was not significant. The relationship between CS and the movement-discrimination task complexity level was assessed for all students who completed the first 16 levels of the movement-discrimination training: seven dyslexic and five normal (TD) students. The movement-discrimination CSF increased significantly as a function of complexity level for each of the test frequency targets, shown in Figure 2 and Table 3, analyzed using a two-factor ANOVA, levels of complexity × individual student’s mean CSF (averaged across five backgrounds for each test frequency), having df = 15. In the movement-discrimination training group, both dyslexic and normal students’ CS, on average, improved steadily with each increasing complexity level attained (Figure 2, mean increase by test frequency in Table 3). The 0.25 cyc/deg test frequency requires pooling of contrast information over several spatial frequency channels since, as shown by Blakemore and Campbell (1969), there are no luminance-varying spatial frequency channels below 1 cyc/deg, requiring the highest contrast thresholds (lowest CS) for movement-discrimination, shown for both normal (TD) (Figure 2A), and dyslexics, (Figure 2B). Moreover, the lowest contrast thresholds (highest CS), and the largest improvements as complexity level was increased, shown in Table 3, were found for the 1 cyc/deg test pattern, shown by the solid line in Figure 2, for both dyslexic, as found previously (Lawton, 2016), and normal students. The difference between the mean CS for each of the four test frequencies is highly significant (*F*_(3)_ = 50.48, *p* < 0.0001 for dyslexics and *F*_(3)_ = 45.95, *p* < 0.0001 for normal students), supporting the hypothesis that the low spatial frequency test patterns, i.e., 0.25 and 0.5 cyc/deg, are processed differently, using probability summation across higher spatial frequency neural channels, than the higher spatial frequency test patterns, i.e., 1 and 2 cyc/deg, activating one-octave wide spatial frequency channels (Blakemore and Campbell, 1969).

Finding an increased CSF at increasing levels of complexity, thereby increasing: (1) the speed of motion, as shown in Table 2; (2) the width of the background frame of reference (from single to multifrequency backgrounds, see Figure 1C); and (3) the background contrast (activating more parvocellular neurons at higher contrasts; Kaplan and Shapley, 1986), suggests that movement-discrimination training improves the functioning of magnocellular neurons (moving test pattern) relative to the functioning of parvocellular neurons (stationary background), since the stimulus parameters of the test patterns were chosen to maximally activate magnocellular neurons (Lawton, 1989, 2000, 2011). The temporal frequencies that the students could not discriminate the direction of movement before training, and had the highest contrast sensitivities following training were the faster moving test patterns, i.e., the 10 and 13 Hz movement (complexity levels 9–16), shown in Figure 2.

Not only was CS for direction-discrimination increased significantly following movement-discrimination training, but also the time to discriminate the direction of movement was reduced significantly for students who did the movement-discrimination intervention. For example, for the 1 cyc/deg test frequency, the most sensitive test frequency target (see Figure 2), the mean time to complete five threshold measurements decreased significantly as the complexity level increased for both dyslexic and normal students, (*F*_(15)_ = 5.61, *p* = 0.0009), from a mean of 5 min down to 2 min for dyslexic students and 6 min down to 3 min for normal students, as complexity level increased. These results indicate that visual timing deficits in dyslexic and normal students are remediated, since both: (1) the sensitivity to discriminate movement-discrimination increased; and (2) the time required to complete movement-discrimination training decreased as the amount of training increased.

**Figure 3:**
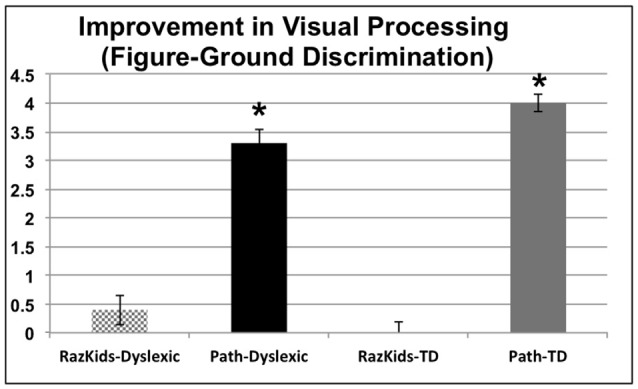
**Improvements in motion direction discrimination after intervention training, each level correlated with Dyslexia Determination Test (DDT) scores of from 6 (above normal) to 1 (markedly below normal)**. *Indicates these improvements were significant, the significance levels being listed in Tables 4A,B.

The variable of Visual Processing Level (VPL), provided by the movement-discrimination diagnostic program which has each participant do the first session of movement-discrimination training, varies between 1: markedly below normal, 2: moderately below normal, 3: mildly below normal, 4: borderline normal, 5: normal in reading up to 6: above normal in reading. Movement-discrimination CS, a measure of visual timing, improved significantly only for those students who were trained on the movement-discrimination intervention, improving in sensitivity 3.3-fold for dyslexic and 4-fold for normal (TD) students after movement-discrimination training, increasing in movement-discrimination from moderately below normal to normal or above normal, based on the DDT classification, whereas those students who were trained on guided reading, both dyslexic and normal, did not improve significantly on movement-discrimination (see Figure 3 and Tables 4A,B), the difference between movement-discrimination training (*PATH*) compared to guided reading (*RK*) training being highly significant (*p* < 0.0008 for dyslexics and *p* < 0.0002 for normal students). The VPL level was analyzed using a mixed factors ANOVA, as above, yielding a main effect of Training, *F*_(1,38)_ = 58.613, *p* < 0.001. Additionally, an interaction occurred between Training and Training Program indicating that students trained on movement-discrimination improved significantly more than did students trained on computer-based guided reading, *F*_(1,38)_ = 35.881, *p* < 0.001, see Figure 3 (an improvement of 3 units, e.g., from 2 to 5 can be thought of as moving from moderately below normal up to normal in reading). No interaction was found with Clinical Group. Follow-up ANOVAs for the computer-based guided reading group alone indicated no significant improvement, *p* > 0.05. Thus, for both dyslexic and normal students, VPL, providing a measure of visual timing, only improved significantly following the movement-discrimination training.

**Table 4B:** **Mean ± SEM values for standardized measures in dyslexic students**.

Skill	Training	Measure	Mean pre	Mean post	Diff	Sig
Motion VPL	*Raz-Kids*	VPL	2.3 ± 0.5	2.7 ± 0.5	0.4	ns
	*PATH*		1.3 ± 0.2	4.7 ± 0.4	3.4	***
Reading rate	*Raz-Kids*	Words/Minute	157.4 ± 17.2	185.7 ± 18.0	28.3	***
	*PATH*		153.0 ± 22.7	219.2 ± 24.1	66.2	***
Comprehension	*Raz-Kids*	GORT %	47.0 ± 5.6	48.2 ± 8.3	1.2	ns
	*PATH*		25.3 ± 7.8	53.3 ± 8.0	28.0	***
Phonological	*Raz-Kids*	CTOPP %	70.7 ± 8.2	65.1 ± 8.0	−5.6	ns
	*PATH*		60.3 ± 7.1	72.5 ± 5.7	12.2	**
Attention	*Raz-Kids*	CAS %	20.2 ± 7.5	33.0 ± 7.7	12.8	*
	*PATH*		26.7 ± 5.6	46.1 ± 6.3	19.4	*
Working memory	*Raz-Kids*	TIPS Visual %	47.3 ± 10.1	53.0 ± 10.3	5.7	ns
		TIPS Auditory %	49.5 ± 8.9	52.4 ± 10.3	2.9	ns
	*PATH*	TIPS Visual %	56.8 ± 8.1	83.2 ± 4.8	26.3	***
		TIPS Auditory %	44.8 ± 7.8	70.6 ± 6.3	25.8	***

Significance (Sig): *p < 0.05, **p < 0.01, ***p < 0.001; Diff, Difference between Mean Pre and Post.

### Improvements in Attention, Reading and Memory

**Figure 4:**
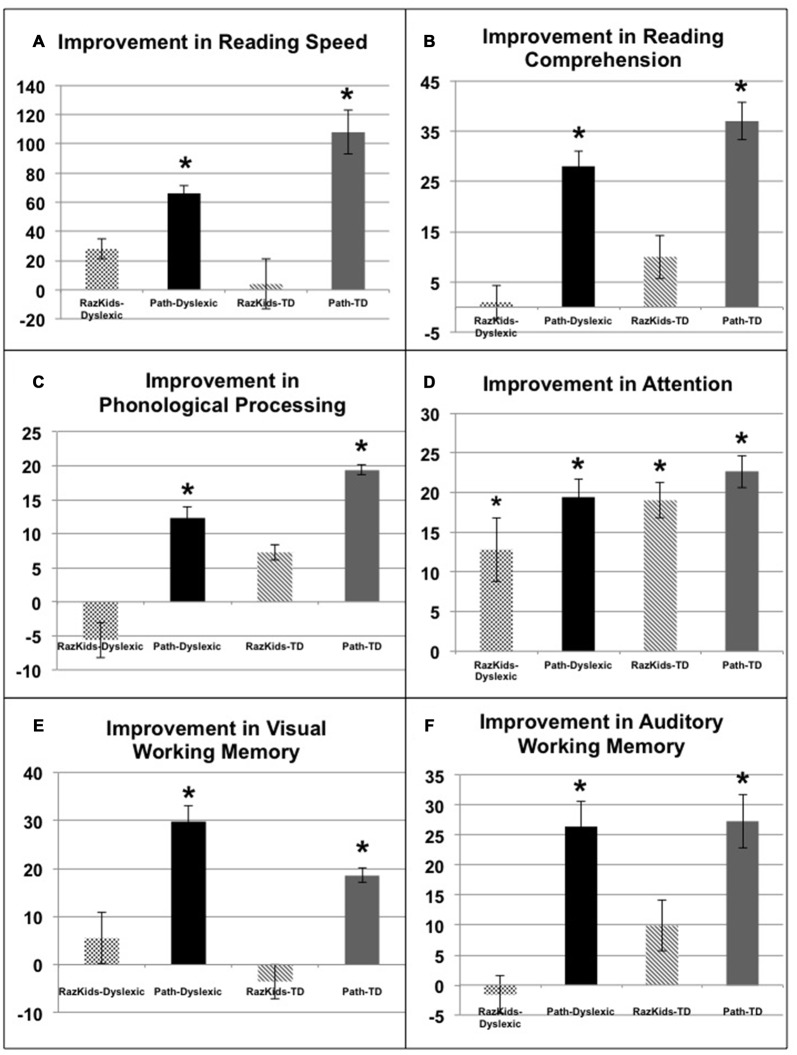
**Improvements in either words/min (A**, Reading Speed) or in standardized percentiles on **(B)** Reading Comprehension (GORT), **(C)** Phonological Processing (CTOPP), **(D)** Attention (CAS), **(E)** Visual Working Memory and **(F)** Auditory Working Memory (TIPS). *Indicates these improvements were significant, the significance levels being listed in Tables 4A,B.

The critical hypothesis to be tested in this study was that movement direction-discrimination training would yield larger Pre-Post gains on the dependent measures than doing the computer-based guided reading program. Additionally, it was necessary to determine whether the effects of training were significant for the Normal (TD) group as well as the Dyslexic group. In order to analyze the effects of training differentially on each group of students, mixed factors ANOVAs were performed with the between subjects factors of Training Group (movement-discrimination vs. guided reading), and Reading Level (Normal vs. Dyslexic) and the within subjects factor of Training (Pre vs. Post). One analysis was performed for each standardized test described above, the mean pre- and post-results shown in Figure 4 and Table 4A (normal) and Table 4B (dyslexic).

*Reading speed*, as assessed by the Computer-Based Reading Speed test, not limited by the student’s rate of speaking, was analyzed by running an ANOVA. All improvements in Reading Speed showed a significant main effect of Training, *F*_(1,38)_ = 17.863, *p* < 0.001, but an interaction between Training and Training Group also was found indicating that the *PATH* movement-discrimination group gained significantly more than did the computer-based guided reading (*RK*) group, *F*_(1,38)_ = 8.363, *p* = 0.006 (Figure 4A and Tables 4A,B). Normal reading rates increased from 219 words/min up to 224 words/min following computer-based reading, an increase of 5 words/min on average, whereas normal reading rates increased from 195 words/min to 303 words/min following movement-discrimination training, an increase of 108 words/min on average, increasing 20-fold more. Dyslexic reading rates increased from 157 words/min up to 186 words/min following computer-based reading, an increase of 29 words/min on average, whereas dyslexic reading rates increased from 153 words/min to 219 words/min following movement-discrimination training, an increase of 86 words/min on average, increasing 3-fold more. Thus, although both normal and dyslexic readers improved significantly in reading comprehension following movement-discrimination training, neither dyslexic nor normal readers improved significantly in reading comprehension following the computer-based guided reading intervention.

*Reading Comprehension*, as assessed by the Gray Oral Reading Comprehension Standard Score Percentile, was then analyzed using an ANOVA. All improvements in Comprehension showed a significant main effect of Training, *F*_(1,38)_ = 27.046, *p* < 0.001, but an interaction between Training and Training Group also was found, indicating that the *PATH* movement-discrimination group gained significantly more than did the computer-based guided reading (*RK*) group, *F*_(1,38)_ = 13.548, *p* = 0.001 (Figure 4B and Tables 4A,B). Normal reading comprehension increased from 51% up to 61% following computer-based reading, an increase of 10% on average, whereas normal reading comprehension increased from 41% to 78% following movement-discrimination training, an increase of 37% on average, increasing 4-fold more. Dyslexic reading comprehension increased from 47% up to 48% following computer-based reading, an increase of 1% on average, whereas dyslexic reading rates increased from 25% to 53% following movement-discrimination training, an increase of 28% on average, increasing 28-fold more. Thus, although both normal and dyslexic readers improved significantly in reading comprehension following the movement-discrimination training, neither dyslexic nor normal readers improved significantly in reading comprehension following the computer-based guided reading intervention.

The reading-related variable of *Phonological Ability* (Figure 4C and Tables 4A,B) was tested using the scores from the CTOPP Blending Words Subtest, the ANOVA revealing a main effect of Training, *F*_(1,38)_ = 9.598, *p* = 0.004. Interestingly, this measure also showed an interaction with Training Group, *F*_(1,38)_ = 7.770, *p* = 0.008. Normal phonological ability increased from 53% up to 61% following computer-based reading, an increase of 8% on average, whereas phonological ability increased from 52% to 72% following movement-discrimination training, an increase of 20% on average, increasing 2.5-fold more. Dyslexic phonological ability decreased from 71% down to 65% following computer-based reading, a decrease of 6% on average, whereas dyslexic phonological ability increased from 60% to 73% following movement-discrimination training, an increase of 13% on average, increasing 19-fold more. A follow-up analysis revealed that only the students trained on movement-discrimination improved significantly in phonological awareness following the intervention training (*RK*, *p* > 0.05).

*Attention*, as measured by the Standardized Percentile Score on the Cognitive Assessment System subtests (Stroop and Number Detection) when analyzed using an ANOVA showed a significant main effect of Training, *F*_(1,38)_ = 44.787, *p* < 0.001. No other significant effects were found, indicating that training was effective regardless of intervention or clinical population. Attention improved significantly for normal students following both reading interventions (Figure 4D and Tables 4A,B). Normal student’s ability to direct attention increased from 33% up to 52% following computer-based reading, an increase of 20% on average, whereas normal student’s ability to direct attention increased from 23% to 45% following movement-discrimination training, an increase of 22% on average, increasing about the same amount. Dyslexic student’s ability to direct attention increased from 20% up to 33% following computer-based reading, an increase of 13% on average, whereas dyslexic student’s ability to direct attention increased from 27% to 46% following movement-discrimination training, an increase of 19% on average. Thus the mean data suggest that Dyslexic students’ attention benefits more from movement-discrimination training, but this claim cannot be supported statistically.

The variable of *Working Memory* was tested by the TIPS visual and auditory modalities sub-scores. The ANOVA revealed that both the visual (*F*_(1.38)_ = 12.748, *p* = 0.001) and auditory (*F*_(1.38)_ = 11.353, *p* = 0.002) modalities (Figures 4E,F and Tables 4A,B) demonstrated main effects of training. These variables also showed significant interactions with the Training group, indicating that movement-discrimination participants increased significantly more than did computer-based guided reading participants (Visual: *F*_(1,38)_ = 9.645, *p* = 0.004); Auditory: *F*_(1,38)_ = 4.301, *p* = 0.045). As seen in Table 4A, *normal visual working memory* decreased from 61% down to 59% following computer-based reading, a decrease of 2% on average, whereas normal visual working memory increased from 68% to 91% following movement-discrimination training, an increase of 23% on average, increasing 12-fold more. *Dyslexic visual working memory* (Table 4B) increased from 47% up to 53% following computer-based reading, an increase of 6% on average, whereas dyslexic visual working memory increased from 57% to 83% following movement-discrimination training, an increase of 26% on average, increasing 4-fold more. *Normal auditory working memory* increased from 52% up to 62% following computer-based reading, an increase of 10% on average, whereas normal auditory working memory increased from 53% to 79% following movement-discrimination training, an increase of 26% on average, increasing 3-fold more. *Dyslexic auditory working memory* increased from 49% up to 52% following computer-based reading, an increase of 3% on average, whereas dyslexic auditory working memory increased from 45% to 71% following movement-discrimination training, an increase of 26% on average, increasing 8-fold more. These results show that only students trained on movement-discrimination improved significantly in both visual and auditory working memory.

The more students improved on movement direction-discrimination, the more they improved in high level cognitive skills, Reading Comprehension being the primary factor. A linear regression was performed on the independent variable of CS (as in Table 3), using the cognitive tests listed in Tables 4A,B (below). All of these dependent measures produced positive correlations with CS, with scores on the CTOPP (*r* = 0.353, *p* = 0.011), and the Reading Comprehension measure (*r* = 0.456, *p* = 0.001) yielding significance. However, the regression revealed that changes in Reading Comprehension alone explained a significant amount of variance in CS, *β* = 0.472, *r*^2^ change = 0.208, *F*_(1,40)_ = 10.527, *p* = 0.002. Normal students commented to our staff at the end of this study that before doing movement direction-discrimination training, they had to spend much effort to read, pay attention and remember, being at risk for reading problems, that following this training was now being done automatically. These results demonstrate that for both dyslexic and normal students, movement-discrimination training, designed to optimally activate magnocellular neurons relative to parvocellular neurons at low levels in the dorsal stream, significantly improved high-level cognitive functions, including reading speed and comprehension, phonological processing, as well as visual and auditory working memory, more than was found following computer-based guided reading. Consequently, this data shows that for both dyslexic and normal students improving dorsal stream function improved attention, reading fluency and working memory, whereas doing guided reading only improved attention.

## Discussion

This study found that only following movement-discrimination training, that is designed to optimally activate magnocellular neurons (Lawton, 1989, 2000, 2011; moving test pattern) in the V1-MT network at low levels in the dorsal stream, relative to parvocellular neurons (stationary background pattern), requiring figure-ground discrimination, not only does the timing and sensitivity of figure-ground discrimination improve, but also the high-level cognitive functions of attention, reading fluency, and working memory all improved significantly for both dyslexic and normal students. The more students improved on movement direction-discrimination, the more they improved in high level cognitive skills, Reading Comprehension being the primary factor. These findings indicate that improving low-level dorsal stream function using this movement figure-ground discrimination paradigm is important for developing high-level cognitive functions like reading fluency for both dyslexic and normal students. This suggests that this type of movement-discrimination training should be provided for all students in grades 2–4, being between 7 and 9 years old, especially since it can be done rapidly, only two times/week for 12 weeks, to provide significant improvements in the executive functions of attention, reading and working memory.

### Visual Timing Deficits Limit Reading Acquisition in Dyslexia

Our working hypothesis is that sluggish magnocellular neurons early in the dorsal cortical visual pathway (V1-MT), causing visual timing deficits that are found in dyslexics (Livingstone et al., 1991; Lehmkuhle et al., 1993), disrupt processing at higher levels of dorsal stream processing, dyslexics having little or no activity in MT (Eden et al., 1996; Demb et al., 1998), including the development of these processes. This study shows that following movement-discrimination training, using patterns optimal for activating the V1-MT network (Allman et al., 1985; Hupé et al., 1998; De Valois et al., 2000; Nassi et al., 2006), these visual timing deficits are remediated for both dyslexic and normal students, causing attention, reading fluency and working memory, all high-level cognitive functions, to improve significantly. These results were found previously in dyslexics, e.g., Lawton (2016), and substantiated by brain imaging studies. After a short amount of movement-discrimination training in dyslexic fourth graders, 3 times/week for 6 weeks, dorsal stream activity improved as shown in Visual Evoked Potentials (Shelley-Tremblay et al., 2011), consistent with a recent pilot study using magnetoencephalography (MEG) source imaging (Lawton and Huang, 2015) that found improved function in both the dorsal stream (V1, V3, MT, MST areas) and fronto-parietal attention networks following 8 weeks of movement-discrimination training twice a week.

Visual timing deficits resulting from sluggish magnocellular (motion-sensitive) neurons in the dorsal stream are likely to be highly involved in the reading deficits of those with dyslexia (Stein and Walsh, 1997; Vidyasagar, 1999, 2001; Lawton, 2000, 2007, 2011, 2016; Stein, 2001). Convergent evidence is consistent with the suggestion of a relationship between visual dorsal stream processing and reading ability, such that poor dorsal stream processing relates to slower timing being assessed by high motion thresholds: dyslexics have been found to have deficits in motion perception at: (1) the retinal level (Tyler, 1974) when measured using the frequency doubling illusion (Buchholz and McKone, 2004; Kevan and Pammer, 2008, 2009; Gori et al., 2014; Avellis et al., 2016); (2) V1 measured using VEPs (Livingstone et al., 1991; Lehmkuhle et al., 1993; Shelley-Tremblay et al., 2011); (3) V1 and MT using both fMRI brain imaging (Eden et al., 1996; Demb et al., 1998) and psychophysical tasks of movement discrimination relative to a stationary background (Lawton, 2000, 2007, 2011, 2016); (4) MT using motion coherence for direction-discrimination (Cornelissen et al., 1995; Talcott et al., 1998, 2000; Hansen et al., 2001; Ridder et al., 2001; Boden and Giashi, 2007; Nicholson and Fawcett, 2007; Boets et al., 2011); (5) anterior cortical areas activated by saccades, i.e., the LIP and Frontal Eye Fields (FEF), found when dyslexics do saccade and antisaccade training tasks (Fischer, 2012); and (6) parietal structures, prefrontal language systems, cerebellum, and basal ganglia (Nicholson and Fawcett, 2007), and poor reading skills (Stein and Walsh, 1997; Vidyasagar, 1999, 2001, 2012; Lawton, 2000, 2007, 2016; Stein, 2001; Boden and Giashi, 2007). Claiming that there is no evidence that visual deficits cause dyslexia (American Academy of Pediatrics 2009) is not a valid claim, since convergent evidence shows that poor visual dorsal stream functioning is associated with poor reading skills, with a previous review (Boden and Giashi, 2007) providing substantial evidence that magnocellular visual deficits contribute to reading problems.

### Figure-Ground Discrimination Framework, Mediated by V1-MT Network, Is Required

Patterned backgrounds, as opposed to featureless backgrounds, require figure-ground discrimination, suggesting that a core deficit in dyslexia may be figure-ground discrimination analyzed by the dorsal stream, consistent with the dyslexic’s deficits: (1) being primarily due to deficits in the spatiotemporal parsing of the letter stream (Vidyasagar, 1999, 2001, 2012), normally transmitted both by feedforward magnocellular (low-contrast movement) input, and from feedback at the attended location from lateral inferior-parietal (LIP) to medial temporal (MT; Saalmann et al., 2007) and from MT to V1 (Hupé et al., 1998); and (2) in excluding noisy backgrounds (Sperling et al., 2006; Benassi et al., 2010). Training with the stationary background frame of reference provided by single and multifrequency backgrounds improves the dyslexic reader’s ability to discriminate the direction of movement, most likely by taking advantage of MT’s center-surround organization (Allman et al., 1985) to facilitate figure-ground discrimination, enabling the dyslexic to improve in reading fluency and attend to wider regions of space.

When stationary backgrounds were not used, e.g., a flickering 2-D noise pattern (Sperling et al., 2006), activating sluggish magnocellular instead of parvocellular neurons, then motion discrimination sensitivity was reduced in dyslexics, whereas when periodic stationary backgrounds were used, as in this study, then motion discrimination sensitivity increased for all students. Previous results (Lawton, 1985, 1989, 2011, 2016 support the hypothesis that multifrequency backgrounds confer an advantage when discriminating the direction of motion, by providing a wider, more structured frame of reference. Even though the dorsal stream consists of predominantly magnocellular neurons, there is input to the dorsal stream from parvocellular neurons (Maunsell et al., 1990; Callaway, 1998; Nassi et al., 2006) from the lateral geniculate nucleus (lgn), V1, and V4, all projecting to MT, enabling parvocellular activity to provide a background frame of reference for discriminating the direction of movement in the dorsal stream.

After movement-discrimination training, the highest contrast sensitivities were found for patterns moving from 10 Hz to 13 Hz, as found in this study and previously (Lawton, 2016), these temporal frequencies being key to improving attention and reading fluency in dyslexics. These results contradict Goswami’s (2011) temporal sampling framework theory, proposing that the key timing deficits in dyslexia are for movement <10 Hz. This study found that improving visual motion direction-discrimination sensitivity and timing (low-levels in dorsal stream) improved processing in the neural networks at high levels of cognitive processing, those mediating attention, reading, and working memory in both normal and dyslexics. These improvements are found by presumably improving low levels in the dorsal stream, the V1-MT network, which improved functioning at higher levels in the dorsal stream, including the PPC, the DLPFC, and the attention networks, as found previously from an MEG brain imaging study (Lawton and Huang, 2015).

This study provides additional evidence that *visual* motion processing is fundamental for paying attention, good reading performance, and remediating reading deficits, contrary to common practice based on the assumption that only auditory-based phonological processing can be used to remediate reading deficits (Tallal, 1980; Tallal et al., 1993; Shaywitz, 2003; Temple et al., 2003; Vellutino et al., 2004; Dehaene, 2009; Olulade et al., 2013). Moreover, the more students improved on movement direction-discrimination, the more they improved in high level cognitive skills, especially in Reading Comprehension. By remediating visual timing deficits in the dorsal stream, improving reading, this suggests a causal role of visual movement-discrimination training and attention in reading acquisition. This study: (1) supports the hypothesis that faulty timing in synchronizing the activity of magnocellular with parvocellular visual pathways in the dorsal stream is a fundamental cause of dyslexia; (2) argues against the assumption that reading deficiencies in dyslexia are caused by phonological or language deficits; and (3) demonstrates that visual movement discrimination is not only a correlate of dyslexia for children and normal students at-risk for reading problems early, but also for its successful treatment. Therefore, a paradigm shift for the treatment of dyslexia from improving phonological processing to improving visual figure-ground movement-discrimination is needed.

### Remediating Visual Timing Deficits Improves High-Level Cognitive Functions

Previously (Lawton, 2016), we found that *only* visual movement-discrimination training as compared to phonological training, either by improving auditory timing (*FastForWord*) or word building strategies (*Learning Upgrade*), when followed by guided reading in the classroom significantly improved both low and high level cognitive functions: (1) motion direction sensitivity; (2) speed of processing for both motion direction-discrimination and reading rates; (3) attention; (4) reading comprehension; (5) phonological processing; and (6) both auditory and visual working memory, including delayed recall. These results indicate that movement-discrimination training improves the sensitivity and timing of sluggish magnocellular neurons (improving dorsal stream function), relative to parvocellular neurons early in the dorsal stream, as evidenced by improved movement-discrimination sensitivity at higher background contrasts and temporal frequencies following movement-discrimination training.

The significant improvements in attention and working memory following movement-discrimination training suggests that training early in the visual dorsal stream improved higher levels of processing in the dorsal stream, in particular the PPC where: (1) selective endogenous attention is encoded (Posner et al., 1984; Posner and Petersen, 1990; Supekar and Menon, 2012), the PPC projecting to the DLPFC, where working memory is encoded (Menon and Uddin, 2010); and (2) spatial attention has been demonstrated to feedback to early visual cortical areas (Watanabe et al., 1998; Martínez et al., 1999; Somers et al., 1999). Moreover, since both phonological processing and auditory working memory improved following visual movement-discrimination training, these improvements demonstrate that visual movement-discrimination training improves auditory skills, suggesting this neurotraining improves the PPC, where a convergence of both auditory and visual inputs in the parietal cortex have been found (Farah et al., 1989). Moreover, the control of spatial attention in early visual cortex is likely directed by regions of the PPC and DLPFC (Silver et al., 2005; Somers, 2014). By improving attention, students were able to hear the sequential ordering of sounds more accurately, improving phonological processing and auditory working memory (Lawton, 2016). Students given training aimed at auditory magnocellular function, as embodied by the *FastForWord* program, improved in reading fluency, but the improvements were not significant when compared to the improvements made by controls, as found in a review of *FastForWord* studies (Strong et al., 2011).

When reading, it has been proposed that the PPC uses the spatial information of the location and overall shape and form of a word it receives through the rapid magnocellular pathway to gate the information going into the temporal stream (Vidyasagar, 1999, 2001). The information is gated via attentional feedback to the striate cortex and to other regions in the occipito-temporal cortex (Watanabe et al., 1998; Martínez et al., 1999; Somers et al., 1999; Vidyasagar, 1999, 2001, 2013), most likely by top-down feedback which uses synchronized neuronal oscillations at the lower end of the gamma frequency range (Vidyasagar, 2013), which can then be used by parvocellular neurons in the ventral stream as a starting point for deciphering the individual letters (Vidyasagar, 1999, 2013). Each cycle of gamma oscillation focuses an attentional spotlight on the primary visual cortical representation of just one or two letters before sequential recognition of these letters and their concatenation into word strings (Vidyasagar, 2013). The timing, period, envelope, amplitude and phase of the synchronized oscillations modulating the incoming signals in the striate cortex have a profound influence on the accuracy and speed of reading (Vidyasagar, 2013). The speed determined by the gamma frequency oscillation is the essential rate-limiting step in dyslexia (Vidyasagar, 2013). Figure/ground movement discrimination training is likely to strengthen coupled: (1) theta/gamma activity for the test patterns moving at 6.7 Hz and 8 Hz; and (2) alpha/gamma activity for the test patterns moving at 10 Hz and 13.3 Hz. Therefore, it is likely that the visual movement-discrimination training paradigm used in this study improves not only magnocellular function and attention, but also magno-parvo integration, figure/ground discrimination, and low gamma frequency oscillation.

The sluggish magnocellular neurons in dyslexics not only result in attention deficits, an impairment in the low gamma frequencies reducing feedback in visual cortical areas (Vidyasagar, 2013), but also disrupted processing in lateral intraparietal cortex (LIP) and FEF, either within a fixation, between fixation sequences, or both (Vidyasagar, 1999; Slaghuis and Ryan, 2006; Fischer, 2012). Moreover, finding that movement-discrimination training improved not only reading fluency, but also attention and working memory indicates that movement-discrimination training helps develop the attention and executive control networks, providing more evidence that abnormal visual motion processing is a fundamental cause of reading and attention problems in dyslexia. By improving the attention network’s functioning, movement-discrimination training provides a wider usable field of view so that more objects are perceived in their correct location in a single glance (Lawton and Stephey, 2009). Movement direction-discrimination training improves the ability to detect synchronicity of multiple objects in space and their trajectories over time, most likely by increasing the ease of magno-parvo integration, thereby facilitating figure-ground discrimination within a wider window of focused attention (Lawton, 2016). Moreover, there is evidence that improvements in reading speed after movement-discrimination training are sustained over time (Lawton, 2011), whereas improvements in word reading found following auditory interventions to improve phonological processing degrade over time, 2 years later showing no difference in word reading compared to controls not having the auditory intervention (Wise et al., 2000). Only when low-level visual timing deficits are remediated are the improvements in high-level cognitive functions, such as reading fluency, sustained over time. When reading, students who allocate all their resources to identify the letters in the word, instead of interpreting a sentence, understanding its meaning, and integrating information into existing knowledge need movement-discrimination training to remediate their visual timing.

In conclusion, visual movement-discrimination training significantly improved both dyslexic and normal students’ selective and sustained attention and visual motion timing and sensitivity, improving figure-ground discrimination, most likely by increasing the temporal precision and neuronal sensitivity of magnocellular neurons relative to linked parvocellular neurons in the dorsal stream. Proper dorsal stream function is essential for reading fluency, selective and sustained attention, and working memory to be done without effort for both normal and dyslexic students. When movement-discrimination training was followed by guided reading in the classroom, attention, reading fluency and working memory skills improved more than found after training on the computer-based guided reading intervention for both dyslexic and normal students. Moreover, the more students improved on movement direction-discrimination, the more they improved in high level cognitive skills, especially in Reading Comprehension. This study suggests that improving visual dorsal stream functioning at low levels by training on figure-ground discrimination of a test pattern moving left or right relative to a stationary background pattern is the key for reading acquisition to happen at an efficient speed for dyslexic and normal students. Remediating visual timing deficits in the dorsal stream revealed the causal role of visual motion discrimination training in reading acquisition. Moreover, this study supports the hypothesis that faulty timing in synchronizing the activity of magnocellular with parvocellular visual pathways in the dorsal stream is a fundamental cause of dyslexia and argues against the assumption that reading deficiencies in dyslexia are caused by phonological or language deficits. This study indicates that a paradigm shift in treating dyslexia from phonologically-based to visually-based methods is essential. Furthermore, this study shows that visual movement direction-discrimination can be used to not only diagnose dyslexia early, but also for its successful treatment, so that reading and learning can be done more automatically, requiring much less effort.

## Author Contributions

TL designed study, recruited and trained staff, ran daily operations and wrote the article. JS-T did all the statistical analyses and helped to write the article.

## Conflict of Interest Statement

The first author TL has a potential conflict of interest, since she is the developer of Path To Reading (PATH), and was employed by Perception Dynamics Institute. The role of this author was to design the study, recruit and train staff, run daily operations and help write the article. She had no part in collecting or analyzing the data, thereby having no influence over the results we obtained. The other author JS-T declares that the research was conducted in the absence of any commercial or financial relationships that could be construed as a potential conflict of interest. The reviewer DC and handling Editor declared their shared affiliation, and the handling Editor states that the process nevertheless met the standards of a fair and objective review.
