# Corrigendum: Training on Movement Figure-Ground Discrimination Remediates Low-Level Visual Timing Deficits in the Dorsal Stream, Improving High-Level Cognitive Functioning, Including Attention, Reading Fluency, and Working Memory

**DOI:** 10.3389/fnhum.2018.00461

**Published:** 2018-11-27

**Authors:** Teri Lawton, John Shelley-Tremblay

**Affiliations:** ^1^Cognitive Neuroscience Research and Remediation, Perception Dynamics Institute, Encinitas, CA, United States; ^2^Department of Psychology, University of South Alabama, Mobile, AL, United States

**Keywords:** dyslexia, perceptual learning, plasticity, timing, reading, attention, memory

In the original article, there was a mistake in Figure 1 as published. The contrast of the figures was too low to be resolved. The corrected Figure 1 appears below. The authors apologize for this error and state that this does not change the scientific conclusions of the article in any way. The original article has been updated.

**Figure 1:**
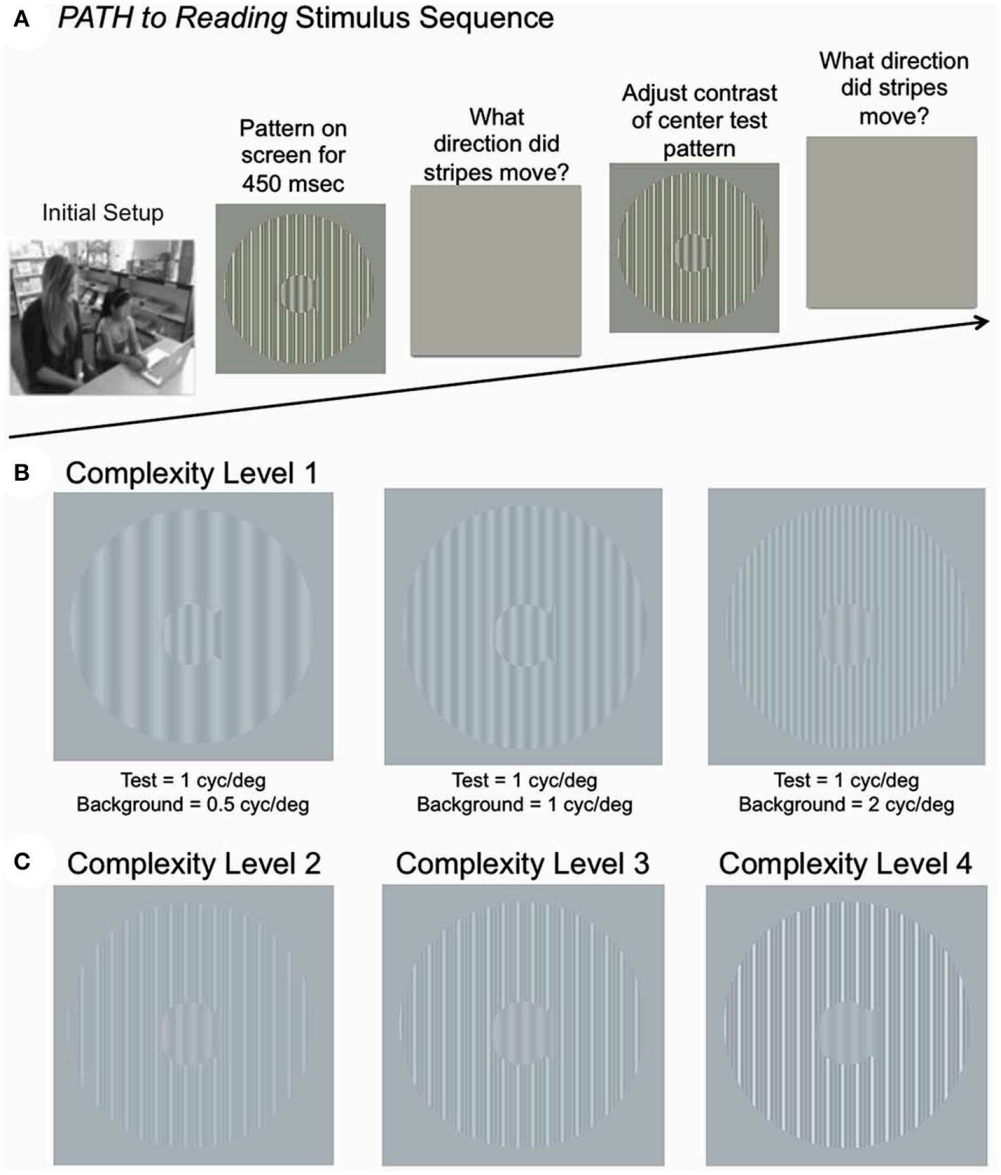
**(A)** Schematic of Stimulus Presentation for *PATH to Reading* intervention. Pattern flashes on screen (shown above) while center stripes move left or right. Screen goes blank, waits for left or right arrow key to be pushed. If incorrect, short tone sounds. Pattern with same or different contrast flashes on screen while center stripes move left or right. Screen goes blank, waits for left or right arrow key to be pushed. This sequence of patterns is presented continuously until the contrast threshold for this pattern is measured. Then the next pattern combination is presented to measure next contrast threshold, until all 20 *PATH to Reading* patterns were presented, and the program says “Thank You” and quits. **(B)** Sample patterns at Complexity Level 1 for a background one octave lower in spatial frequency (0.5 cyc/deg) than the test frequency, equal in spatial frequency to the test frequency (1 cyc/deg), and one octave higher in spatial frequency (2 cyc/deg) than the test frequency for a 1 cyc/deg “fish shaped” test pattern. **(C)** Sample patterns at Complexity Levels 2, 3, and 4 for the center pattern in **(B)**. These patterns have multifrequency background patterns (1 cyc/deg + 2 cyc/deg + 3 cyc/deg) for a 1 cyc/deg test pattern on a 5% (Complexity Level 2), 10% (Complexity Level 3), and 20% (Complexity Level 4) contrast background. These same four complexity levels are repeated at subsequently faster speeds for each set of four complexity levels, increasing from 6.7 Hz (complexity levels 1–4) to 8 Hz (complexity levels 5–8) to10 Hz (complexity levels 9–12) to 13.3 Hz (complexity levels 13–16), as listed in **Table 2**.

## Conflict of interest statement

The authors declare that the research was conducted in the absence of any commercial or financial relationships that could be construed as a potential conflict of interest.

